# Poor Sleep Quality is Linked to Elevated Extracellular Vesicle-Associated Inflammatory Cytokines in Warfighters With Chronic Mild Traumatic Brain Injuries

**DOI:** 10.3389/fphar.2021.762077

**Published:** 2022-01-27

**Authors:** Jackie L. Gottshall, Vivian A. Guedes, Josephine U. Pucci, Daniel Brooks, Nora Watson, Phorum Sheth, Ainslee Gabriel, Sara Mithani, Jacqueline J. Leete, Chen Lai, Bao-Xi Qu, Christina Devoto, Jessica M. Gill, Kimbra Kenney, J. Kent Werner

**Affiliations:** ^1^ Center for Neuroscience and Regenerative Medicine, Uniformed Services University of the Health Sciences, Bethesda, MD, United States; ^2^ National Institute of Nursing Research, National Institutes of Health, Bethesda, MD, United States; ^3^ Department of Neurology, Uniformed Services University of the Health Sciences, Bethesda, MD, United States; ^4^ F. Edward Hebert School of Medicine, Uniformed Services University of the Health Sciences, Bethesda, MD, United States; ^5^ Walter Reed National Military Medical Center, Bethesda, MD, United States; ^6^ United States Naval Academy, Annapolis, MD, United States; ^7^ National Intrepid Center of Excellence, Walter Reed National Military Medical Center, Bethesda, MD, United States

**Keywords:** traumatic brain injury, sleep, inflammation, cytokines, extracellular vesicles, IL-6, IL-10, tNF-alpha

## Abstract

**Background:** Elevations of inflammatory cytokine levels occur immediately after mild traumatic brain injury (mTBI) and can persist for years. These elevations have been associated with neuropsychological outcomes, including depression and PTSD symptoms. Sleep disorders, another common sequelae of mTBI, are independently associated with inflammation in otherwise healthy individuals. However, whether sleep and inflammation are linked in chronic mTBI has not been reported.

**Methods:** A retrospective cross-sectional cohort of warfighters was used to investigate the hypothesis that inflammation may be linked to sleep quality in chronic mTBI. Clinical history, peripheral blood samples, and sleep quality scores were collected from 182 warfighters (*n* = 138 mTBI; *n* = 44 controls) during enrollment in the Chronic Effects of Neurotrauma Consortium study. Biomarkers of inflammation (IL-6, IL-10, TNFα cytokines) from plasma and plasma-derived extracellular vesicles (EVs) were quantified using single molecule array. Relationships between sleep quality and cytokine levels were assessed, controlling for age, sex, and BMI. Using clinical cutoff scores for sleep quality, mTBI patients were then divided into “good” and “poor” sleepers and cytokine levels compared between groups.

**Results:** In mTBI participants, sleep quality was significantly associated with EV levels of IL-10 [ß (SE) = 0.11 (0.04), *p* = 0.01] and TNFα [ß (SE) = 0.07 (0.03), *p* < 0.01]. When divided according to “good” versus “poor” sleepers, those reporting poor sleep had significantly elevated EV IL-10 compared to those reporting good sleep [ß (SE) = 0.12 (0.04), *p* < 0.01]. Plasma-derived associations were not significant. No associations were found between sleep quality and cytokine levels in controls.

**Conclusion:** These results suggest a significant relationship between sleep quality and chronic inflammation in mTBI patients. Clinically, mTBI patients with a high likelihood of sleep disorders demonstrate elevated levels of inflammatory cytokines. Signal from EVs, though smaller in magnitude, may have stronger clinical associations than from plasma. Sleep-focused interventions may also serve to regulate chronic inflammatory processes in these patients. Larger prospective studies are needed to investigate the mechanisms and therapeutic implications of the likely bi-directional relationship between sleep and inflammation following mTBI.

## Introduction

Sleep complaints are some of the most common long-term sequelae of mild traumatic brain injury (mTBI), affecting approximately 50% of mTBI-exposed individuals (Mathias and Alvaro, 2012). The vast majority of these individuals describe insufficient and disturbed sleep post-mTBI (Collen et al., 2012; Wickwire et al., 2018) and many are ultimately diagnosed with a variety of sleep disorders including insomnia, circadian dysregulation, fatigue, hypersomnolence, obstructive sleep apnea, or narcolepsy (Mathias and Alvaro, 2012; Wickwire et al., 2018). Accumulating evidence suggests that poor sleep may exacerbate long-term sequelae across functional domains, resulting in increased risk of poor mental and physical health outcomes (Mathias and Alvaro, 2012; Theadom et al., 2015; Wickwire et al., 2018). Severe TBI patients with sleep-wake cycle disturbances average significantly longer post-injury stays at both initial trauma centers and secondary rehabilitation facilities than those without sleep-related disturbances (Makley et al., 2008). Biomarker findings in chronic mTBI patients demonstrate that poor sleep quality is correlated with increased levels of tau and neurofilaments, which are associated with poor neurodegenerative outcomes including Alzheimer’s disease and Chronic Traumatic Encephalopathy (Lucke-Wold et al., 2015; Wickwire et al., 2018; Werner et al., 2021). Sleep disturbances are also known to exacerbate common neuropsychiatric symptoms post-mTBI including executive dysfunction (Lucke-Wold et al., 2015), emotional and mood disturbances, depression, PTSD, and chronic pain (Wickwire et al., 2018). Despite the growing body of literature that supports a relationship between sleep quality and functional outcomes following mTBI, the pathophysiology behind this relationship remains unclear.

Interestingly, both TBI and sleep dysfunction are independently associated with inflammation (Vgontzas et al., 2004; Gill et al., 2018; Sun et al., 2019; Vedantam et al., 2021). Acutely after TBI, pro- and anti-inflammatory cytokines, such as interleukin-6 (IL-6), interleukin-10 (IL-10), and tumor necrosis factor alpha (TNFα), are elevated for days to months before declining (Witcher et al., 2015; Sun et al., 2019). These cytokines reach peak levels 4–12 h after the initial trauma but appear to have more persistent elevations with repeated mTBIs (Arand et al., 2001; Cheng et al., 2019). Though mechanisms are unclear, chronically symptomatic TBI patients also have high levels of inflammatory cytokines and are more likely to experience poor outcomes 1 year post-injury, including memory failure and other cognitive impairments (Theadom et al., 2015; Sun et al., 2019). Similar alterations of inflammatory cytokines have also been reported in healthy individuals following short- and long-term sleep deprivation. In a cohort of 12 healthy male participants, Chennaoui and colleagues (Chennaoui et al., 2011) found that one night of acute sleep deprivation induced significant increases in TNFα levels. Shearer et al. (2001) similarly reported that four nights of total sleep deprivation resulted in significant increases in levels of IL-6 and soluble TNFα receptor I in 21 healthy individuals. These inflammatory processes extend into chronic timescales as well, such that IL-6 and TNFα levels are correlated with severity of self-reported sleep dysfunction in otherwise healthy individuals with chronic insomnia (Ren et al., 2021).

The presence of strikingly similar cytokine modulations in populations with mTBI and with sleep dysfunction invites the question of whether sleep and inflammation may be bidirectional processes that influence recovery in mTBI patients. Such bidirectional modulation has precedence in the non-TBI brain. Acute enhancement of TNFα has been demonstrated to promote sleep (Rockstrom et al., 2018) and is also induced by chronic sleep restriction (Burgos et al., 2006). Sleep restriction has similarly been shown to induce IL-6 production (Burgos et al., 2006), while IL-10 has been demonstrated to suppress non-REM sleep (Kushikata et al., 1999). These pathways may be modulated by common regulatory molecules, such as adenosine, which is a critical component of both the sleep-wake cycle (via sleep induction) and immunoregulation (via inhibition of inflammatory cytokines, including TNFα) (Haskó and Cronstein, 2013; Reichert et al., 2014; Cekic and Linden, 2016; Ohta, 2016). However, the connections between sleep and inflammatory processes have not been examined in mTBI patients. If these processes are linked post-mTBI, it would have important clinical implications for the utility of sleep as an accessible therapeutic entry point for the regulation of chronic inflammatory processes and potential improvement of long-term outcomes. Furthermore, it may implicate inflammatory pathway targets for therapy in mTBI-related sleep disorders.

Regarding biomarker isolation, while plasma and serum are common sources, extracelluluar vesicles (EVs) have attracted increasing attention in immunological, oncological, and neurological pathologies, including TBI (Kenney et al., 2018; Goetzl et al., 2019; Guedes et al., 2020a; Guedes et al., 2020b). Cytokines can be released either in free (soluble) forms or bound to/within EVs (Zhang et al., 2006; Konadu et al., 2015; Sass et al., 2020), however, the relative significance of cytokines derived from the two sources is not established. EVs are small vesicles released by cells and formed by a lipid bilayer containing cargo that includes proteins, lipids, and RNAs. Depending upon their biogenesis, EVs can be broadly divided into exosomes and larger “microvesicles” (Colombo et al., 2014; van Niel et al., 2018). Although the role of EVs in physiological and pathological processes is only beginning to be understood (van Niel et al., 2018; Margolis and Sadovsky, 2019), growing literature supports EV secretion as a form of cell-to-cell communication, perhaps via targeted delivery. EV-encapsulated cytokines have been demonstrated to be biologically active and capable of interacting with other cells (Fitzgerald et al., 2018). The EV membrane protects its contents against enzymatic degradation, and its cytokines may be of greater physiologic relevance than those in the plasma, rendering them promising biomarker sources with potential for a superior signal-to-noise ratio (Mustapic et al., 2017; Fitzgerald et al., 2018; Guedes et al., 2020b; Chung et al., 2020).

The present study sought to examine whether self-reported sleep quality was associated with inflammatory cytokine levels (IL-6, IL-10, TNFα) in warfighters with a history of mTBI. Cytokine levels were measured in both plasma and plasma-isolated EVs for each subject. In addition to utilizing full self-report scales as a graded measure of sleep quality, cytokine levels were also analyzed according to the categorical presence or absence of clinically relevant sleep complaints, facilitating contextualization in terms of clinical practice.

## Methods

### Study Population

The study population consists of a cross-sectional retrospective convenience sample of combat-exposed US military servicemembers and veterans, termed “warfighters.” Lifetime mTBI history, blood draws, and neuropsychological assessments were collected from 182 participants during baseline enrollment in the ongoing Chronic Effects of Neurotrauma Consortium (CENC) multi-site observational study (now LIMBIC-CENC under continuing funding). Participants were enrolled between 2015 and 2016 according to CENC protocol and approved by appropriate institutional review boards.

### mTBI Diagnosis

For each participant, mTBI diagnosis was determined based on CENC protocols detailed previously (Werner et al., 2021). Briefly, all underwent detailed structured and unstructured interviews as well as medical history reviews. Structured interviews included an identification of all potential concussive events across the participant’s lifetime using the Ohio State University TBI Identification (OSU TBI-ID) instrument. Each potential concussive event was assessed for preliminary mTBI diagnosis using the Virginia Commonwealth University retrospective concussion diagnostic interview and DOD/VA common mTBI definition. Preliminary mTBI diagnoses were vetted against unstructured interviews and medical documentation pertaining to the event and final diagnoses administered by the site principal investigator (PI). Any diagnostic uncertainty was adjudicated by a central diagnosis committee of national TBI experts. Participants were excluded if TBI was determined to be moderate, severe, or penetrating.

### Biomarker Analysis

#### Extracellular Vesicle Isolation From Plasma Samples

Blood samples were collected using tubes containing ethylene diamine tetraacetic acid (EDTA) from all participants between 8 am and 3 pm and processed within 4 h of collection. All samples were centrifuged at 3000 rpm for 10 min (4°C) and plasma was aliquoted and stored at −80°C until analyzed. For each participant, 0.5 ml of frozen plasma were used to isolate EVs. Thrombin was added to thawed samples, incubated at room temperature for 5–10 min and then centrifuged for 5 min at 10,000 rpm. The supernatant was removed from each sample and ExoQuick (System Biosciences) solution was added according to manufacturer’s instructions. After a 30-min incubation at 4°C, samples were centrifuged at 1,500 g for 30 min. The supernatant was aspirated resulting in an EV pellet at the bottom of the tube. The pellet was resuspended in 500 µL phosphate-buffered saline. We used a human tumor susceptibility gene 101 protein (TSG101, EV marker) ELISA kit to confirm the presence of EVs in the samples (CSBEL025125HU, CUSABIO, Houston, TX) (Supplementary Table S1). For protein analysis, mammalian protein extraction reagent (M-PER) was added to each tube to lyse EVs (Thermo Scientific, Inc., Rockford, IL).

#### Protein Analysis

Plasma and EV Levels of IL-6, IL-10 and TNFα were measured using an ultrasensitive assay (Neurology 3-Plex A, item 101995, Quanterix Corporation, Lexington, MA) and single-molecule technology (SIMOA™) on a HD-1 analyzer (Quanterix Corporation) and reported in pg/mL. Samples were randomized over plates and tested in duplicate with laboratory scientists blinded to participant groups. All samples underwent quality control analysis and samples with reported coefficients of variation over 20% were not used for analysis. Samples with high coefficients of variation and concentrations below lower limits of quantification were replaced by values equal to half of the limit of concentration respective to the assay.

### Sleep Quality Assessment

Sleep quality was assessed with the Pittsburgh Sleep Quality Index (PSQI) self-report questionnaire (Buysse et al., 1989; Carpenter and Andrykowski, 1998). The PSQI is a widely used measure comprised of 19 items, each item scored on a 4-point Likert scale. Items are focused on sleep-related problems and sleep quality, including sleep duration and delay in falling asleep. PSQI scores range from 0 to 21; higher scores indicate increasingly poor sleep quality.

### Statistical Analyses

For all cytokine biomarkers, outliers were removed at a threshold of 5x standard deviation. A conservative threshold of 5x standard deviation was chosen to maximally preserve true data variability while excluding samples that fell outside of the probable physiological range. Data were log transformed to achieve approximate normality. A large percentage of EV protein samples fell below respective lower limits of quantification for all three cytokines; frequencies of these samples were similar in mTBI and control cohorts (Supplementary Table S2). Analyses were performed with and without samples below the lower limit of quantification to evaluate the extent to which assay sensitivity may influence results. Because directionality was unchanged with inclusion or exclusion of below-limit samples, reported analyses include all evaluated samples.

Demographic characteristics were summarized by mean ± SD for continuous variables and counts for categorical variables. Cytokine concentrations were described by median and inter-quartile range. Student’s T-tests were used to compare log-transformed cytokine concentrations between mTBI and control cohorts. Separate analyses were performed for plasma-derived and EV-derived cytokine samples.

Linear relationships between sleep quality (PSQI score) and inflammatory cytokine levels were evaluated separately for mTBI and control cohorts using Spearman’s correlations. Data distribution and correlations were visualized using univariate kernel density estimates and best-fit linear regression lines. Multivariable linear regression models were used to estimate independent associations of sleep quality (PSQI score) with cytokine levels (standardized to mean of zero and standard deviation of one) with adjustment for age, sex, and body mass index (BMI) (i.e., potential confounders expected to influence inflammatory biomarker levels). Multivariable linear regression models were also constructed to evaluate potential interactions between sleep quality and additional predictors, adjusting for age, sex, and BMI. Interactions of cohort with PSQI were tested for each cytokine to evaluate the hypothesized stronger relationship of sleep quality with inflammatory biomarkers in the mTBI versus control cohort. In the mTBI cohort, we additionally evaluated whether there was an interaction effect of number of TBI events and PSQI score on cytokine levels.

Additional multivariable linear regression models were used to evaluate the potential predictive value of categorical sleep quality (“good” vs. “poor”) on cytokine levels, adjusted for age, sex, and BMI. Patients with PSQI <10 were classified as “good” sleepers and those with PSQI ≥10 as “poor” sleepers, according to established guidance on clinically significant sleep complaints in a military population (Matsangas and Mysliwiec, 2018).

## Results

### Demographics and Biomarker Summary

Of 182 participants included in the study, 138 (76%) were in the mTBI cohort and 44 (24%) were healthy controls. The sample was predominantly male (86%) with an average age of 40 years (SD = 10.7) and BMI of 30.3 (SD = 5.4). 70% of participants identified as white, and most (87%) completed at least 1 year of college. In the mTBI cohort, the average number of TBIs was 2.5 (SD = 1.8) with 9 years (SD = 4.3) since the most severe mTBI. Demographic characteristics likely to influence inflammatory biomarker levels (i.e., age, sex, and BMI) were similar between mTBI and control cohorts, regardless of the presence or absence of clinically significant sleep complaints (Table 1).

**TABLE 1:** Population demographics according to presence/absence of mTBI history and clinically significant sleep complaints. PSQI score greater than or equal to 10 was considered clinically significant according to accepted guidelines for military populations.

Characteristic	mTBI		Controls	
	PSQI <10	PSQI ≥10	PSQI <10	PSQI ≥10
Count, n	56	82	26	18
Age, mean (SD)	40.3 (11.2)	39.9 (10.1)	39.7 (13.2)	40.4 (9.0)
BMI, mean (SD)	29.5 (5.6)	31.4 (5.6)	29.1 (4.9)	29.2 (4.5)
Male, % (n)	82.1 (46)	89.0 (73)	84.6 (22)	83.3 (15)

IL-6, IL-10, and TNFα protein concentrations were quantified from each participant using EV and plasma sources (see Supplementary Table S2 for a detailed breakdown of values below the lower limit of quantification for each protein detection assay. Supplementary Table S3 provides a descriptive summary of cytokine levels with and without these values). Overall, EV IL-6 concentration was higher in the mTBI cohort [mean (SD) = 0.70 (0.90) pg/ml] versus controls[(mean (SD) = 0.32 (0.43) pg/ml], *p* = 0.040. No between-group differences were found for plasma IL-6, EV and plasma IL-10 or EV and plasma TNFα concentrations.

### EV Inflammatory Biomarker Levels Are Associated With Sleep Quality in mTBI Patients

**FIGURE 1:**
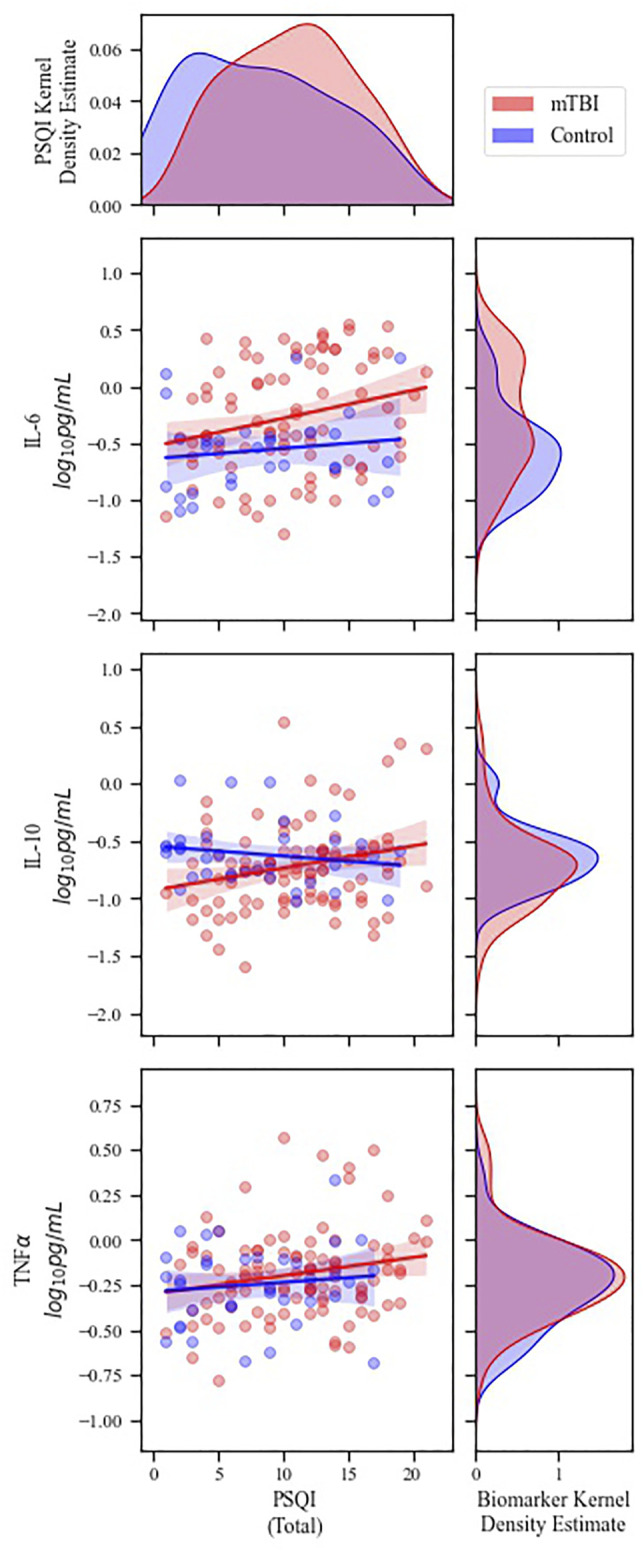
Correlations between PSQI score and EV-derived inflammatory cytokine concentrations. Main plots) Scatterplots of PSQI versus EV cytokine concentrations. Lines represent linear best fit with 95% confidence intervals. Significant correlations were demonstrated between PSQI and all three cytokine levels in the mTBI cohort (see Table 2 for detailed statistics). Marginal plots) Kernel density estimates representing the distribution of PSQI scores (top) and cytokine biomarker concentrations (right) for each cohort. Blue = control patients; Red = mTBI patients. For clarity, data are visualized without values below the lower limits of quantification.

Spearman’s correlations were calculated to determine whether there may be a relationship between sleep quality (PSQI score) and cytokine levels following mTBI; correlation coefficients for each cohort are reported in Table 2. In the mTBI cohort, PSQI was positively correlated with each EV-derived cytokine (Figure 1) and with plasma-derived IL-6 (Supplementary Figure S1). Statistical significance was not reached for any PSQI-cytokine combination in the control cohort. However, a negative association between PSQI and plasma IL-10 approached significance in controls. (Table 2).

**TABLE 2:** Spearman’s correlations for the relationship between PSQI and inflammatory cytokine concentrations in control and mTBI cohorts. + denotes *p* < 0.10; * denotes *p* < 0.05; ** denotes *p* < 0.01.

		EV			Plasma		
		⍴	*p*-value		⍴	*p*-value	
Control	IL-6	0.09	0.60		-0.20	0.27	
	IL-10	-0.19	0.23		-0.27	0.09	+
	TNF⍺	0.00	1.00		-0.17	0.33	
						
mTBI	IL-6	0.20	0.03	*	0.21	0.03	*
	IL-10	0.23	<0.01	**	0.15	0.10	
	TNF⍺	0.22	0.01	*	-0.07	0.45	

To determine whether correlations observed in the mTBI cohort might be partially explained by additional variables expected to influence inflammatory processes, multivariable linear regression models were constructed to estimate the association of PSQI with inflammatory cytokine levels after adjusting for age, sex, and BMI. In these models, PSQI remained associated with higher EV IL-10 and EV TNFα levels in mTBI patients (Table 3). Estimated associations in the mTBI cohort did not substantially vary by patient history of multiple TBI or number of TBI events (Supplementary Table S4).

**TABLE 3:** Regression models estimating the predictive value of PSQI score on cytokine levels (standardized) in mTBI patients. All models control for the effects of age, sex, and BMI. SE represents standard error of the ß coefficient. * denotes *p* < 0.05; ** denotes *p* < 0.01.

Biomarker	EV				Plasma			
	ß	SE	*p-*value		ß	SE	*p*-value	
IL-6	0.09	0.06	0.14		0.07	0.05	0.20	
IL-10	0.11	0.04	0.01	*	0.02	0.04	0.64	
TNF⍺	0.07	0.03	<0.01	**	0.00	0.03	0.85	

For completeness, an additional set of models was calculated to estimate the hypothesized moderating effect of mTBI cohort on the relationship between PSQI and cytokine levels. Overall, PSQI was associated with higher EV IL-10 [ß (SE) = 0.08 (0.04), *p* = 0.04] and TNFα [ß (SE) = 0.06 (0.02), *p* = 0.01] independent of age, sex and BMI (Supplementary Table S5). An association with EV IL-6 approached significance. Interaction terms for PSQI with mTBI were not significant. Beta coefficients for interaction terms ranged from −0.03 to 0.09.

### Clinically Poor Sleepers Have Elevated EV IL-10 Compared With Their Good Sleeper Counterparts

**FIGURE 2:**
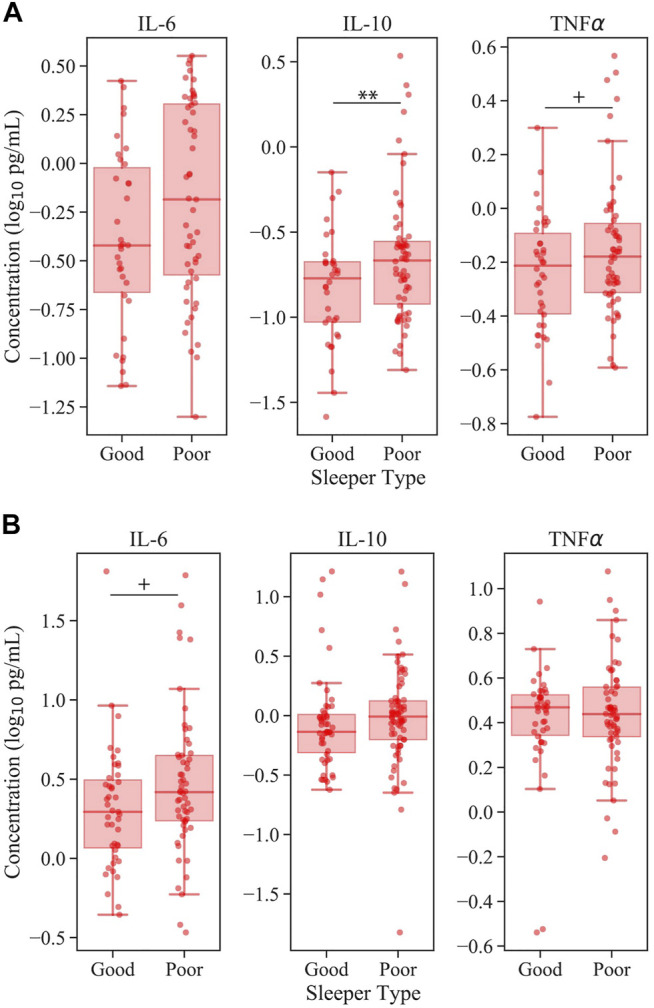
Inflammatory cytokine levels according to sleeper type in mTBI. Panel **(A)** represents EV cytokine levels and panel **(B)** represents plasma cytokine levels. All models control for the effects of age, sex, and BMI. For clarity, data are visualized without values below the lower limits of quantification. Note the significant elevation of EV IL-10 in mTBI patients with poor sleep. + denotes *p* < 0.10; ** denotes *p* < 0.01.

To evaluate clinical relevance of the observed associations between PSQI and cytokine levels in mTBI patients, the mTBI cohort was divided into clinically “good” and “poor” sleeper types according to PSQI score <10 and ≥10, respectively, as has been previously reported in the military population (Matsangas and Mysliwiec, 2018). In linear regression models of the potential predictive value of sleeper type on cytokine level in this cohort, the subset of mTBI patients who reported clinically significant sleep complaints (considered “poor” sleepers) had elevated levels of EV IL-10 compared to mTBI “good” sleepers, after controlling for age, sex and BMI: ß (SE) = 0.12 (0.04), *p* < 0.01 (Figure 2A). Elevations in mTBI poor sleepers approached significance for EV TNFα [ß (SE) = 0.05 (0.027), *p* = 0.08, Figure 2A] and plasma IL-6 [ß (SE) = 0.10 (0.05), *p* = 0.07] (Figure 2B). In these analyses, interactions of number of TBI events with sleeper type were not significant (Supplementary Table S6).

## Discussion

The current study sought to explore whether sleep quality may play a role in chronic inflammatory processes that occur following mTBI. In healthy individuals, sleep and inflammation are bidirectionally regulated (Burgos et al., 2006; Rockstrom et al., 2018). However, the high prevalence of sleep complaints combined with prolonged inflammatory elevations following mTBI (Gill et al., 2018; Vedantam et al., 2021; Werner et al., 2021) suggests that this regulatory feedback loop may be hindered post-injury. Furthermore, although inflammatory signaling is known to occur through both free and extracellular vesicle (EV) associated cytokines, mounting evidence suggests that EV cytokines are particularly promising clinical biomarkers of post-TBI pathophysiologies (Devoto et al., 2016; Mustapic et al., 2017; Gurunathan et al., 2019; Paolicelli et al., 2019; Mondello et al., 2020). Here we confirmed that warfighters with a history of mTBI demonstrate significantly elevated EV IL-6 cytokines compared to their non-TBI counterparts. When considering sleep quality, EV cytokines IL-10 and TNFα were significantly correlated with PSQI score (Table 3; Figure 1), suggesting there may be greater inflammation with worsening sleep quality in mTBI. This effect persisted when mTBI participants were divided according to the presence or absence of clinically significant sleep complaints, such that those at risk for sleep disorders had elevated EV IL-10 compared to those without (Figure 2). No associations were found between sleep quality and plasma cytokines in any model after adjusting for confounds (Table 3; Figure 2B). To our knowledge, this is the first study to establish an association between sleep quality and inflammatory EV cytokine levels in the context of mTBI. These initial findings underscore the importance of addressing clinical sleep complaints – not only for immediate quality of life improvements but also for potential improvement of long-term outcomes following mTBI. They also highlight the need for future prospective studies to establish causality and potential directionality of sleep-immune crosstalk in chronic mTBI.

### Poor Sleep Quality is Associated With Elevation of Pro- and Anti-Inflammatory Cytokines in mTBI Warfighters

Initial exploratory analyses revealed significant associations between sleep quality (PSQI score) and each examined EV-derived cytokine (IL-6, IL-10, TNFα) in the mTBI cohort (Figure 1; Table 2). No associations were found for non-mTBI controls. To determine whether these findings represented a PSQI-cytokine relationship that was truly unique to the mTBI cohort, subsequent analyses were conducted to evaluate whether cohort was a moderator of PSQI-cytokine associations across participants. These analyses did not find cohort to be a statistically significant moderator (Supplementary Table S5). However, we note that the distribution of our dataset was particularly skewed toward the mTBI cohort, with mTBI individuals comprising 76% of the total population (*n* = 138 mTBI and *n* = 44 controls). The standard deviations of ß coefficients within moderator models were also notably large (Supplementary Table S5), suggesting potential statistical underpowering. Larger sample sizes may be needed to definitively support or refute a differential effect of sleep quality according to mTBI history in this population. Given our primary objective of exploring a link between inflammatory cytokines and sleep quality in mTBI, subsequent analyses were narrowed to the mTBI cohort only.

In the mTBI cohort, subsequent regression analyses controlling for age, sex, and BMI demonstrated significant relationships between PSQI and EV-derived pro-inflammatory TNFα, as well as anti-inflammatory IL-10 (Table 3). These findings are consistent with prior studies conducted in otherwise healthy individuals with chronic insomnia. Acutely, sleep deprivation has been shown to result in an increase in pro-inflammatory cytokines and concurrent decrease in anti-inflammatory cytokines (Hirotsu et al., 2012; Taraz et al., 2013). At longer timescales, this trend shifts into an upregulation of both pro-and anti-inflammatory markers (Wright et al., 2015; Ren et al., 2021). In a recent study of 110 individuals with and without chronic insomnia, Ren et al. (2021) demonstrated that both TNFα and IL-10 levels were elevated in insomnia patients, with TNFα positively correlating with PSQI score. Conversely, adenosine levels were found to be reduced and negatively correlated with PSQI. Given the established role of adenosine in both sleep and immune regulation, the authors hypothesized that reduced adenosine signaling may underlie the observed cytokine-PSQI relationships.

Regarding immune regulation in the mTBI brain, only a small number of studies have previously examined inflammatory cytokines in mTBI patients (Gill et al., 2018; Sun et al., 2019; Vedantam et al., 2021). Some studies have reported acute (days to weeks) initial elevations of IL-6 that dissipate over the subsequent 3–6 months (Sun et al., 2019; Vedantam et al., 2021). Here we show that IL-6 levels were significantly elevated at an average of 9 years since mTBI (*p* = 0.40). Since IL-6 production is induced by sleep restriction (Burgos et al., 2006), this finding may be indicative of a delayed secondary IL-6 upregulation associated with chronic sleep deprivation following mTBI. In support of this interpretation, sleep complaints were more common in the mTBI cohort; 59% of mTBI patients reported a PSQI ≥10 compared to 40% of controls (Table 1). Whether IL-6 remains high in some individuals or falls and rises again over the following years is unclear. Prospective studies tracking sleep quality and cytokine levels longitudinally after mTBI will be needed to characterize these interactions.

Previous studies have also found prolonged elevations of IL-10 following mTBI, which have been proposed to provide anti-inflammatory signaling in an attempt to counteract pro-inflammatory responses to injury (Gill et al., 2018; Vedantam et al., 2021). While absolute levels of IL-10 were not upregulated in our mTBI cohort compared to controls, they were significantly associated with poor sleep quality in mTBI (Figure 1; Tables 2, 3). Potentially underlying this association, IL-10 has been shown to inhibit the release of sleep-promoting regulatory molecules and to suppress non-rapid eye movement sleep in animal models (Kanaan et al., 1998; Dugas et al., 1998; Kushikata et al., 1999). One such regulatory molecule, adenosine, has potential for mechanistic parsimony, explaining the increase of both IL-10 and TNFα with worsening sleep quality as demonstrated previously in chronic insomnia (Ren et al., 2021) and now also in mTBI (Figure 1; Table 3). This potential mechanism is further supported by an extensive body of literature surrounding adenosine modulation in response to TBI (Lusardi, 2009; McGuire et al., 2019) and adenosine 2A receptors as critical components of sleep initiation (Lazarus et al., 2019; Ren et al., 2021). Future studies will be needed to explore this hypothesis further.

### IL-10 is Significantly Elevated in mTBI Warfighters With Increased Risk of Sleep Disorders

We additionally sought to contextualize the demonstrated associations between sleep quality and cytokine levels according to the clinical presentation of sleep complaints in mTBI participants. The mTBI cohort was divided into “good” and “poor” sleepers according to whether reported PSQI score was below or above the diagnostic threshold for identification of clinical sleep difficulties (Matsangas and Mysliwiec, 2018). According to this cut-off, the “poor” sleepers were expected to represent a group that would be naturally stratified within the current standard of care. Using this stratification, we found that mTBI patients who report poor sleep demonstrated significantly elevated EV IL-10, with trending significance for TNFα elevation (Figure 2A). These findings suggest that inflammatory alterations associated with sleep difficulties are not confined to subclinical gradations but rather can be inferred at the clinical level.

The finding that cytokine levels differ according to the presentation of sleep complaints is especially relevant to clinical outcomes as increasing evidence demonstrates that immune dysregulation has the potential to cause neurodegenerative disease [see Verboon et al. (2021) for a brief review]. We have previously shown that poor sleep is linked to elevated biomarkers of neurodegeneration in this same cohort of mTBI patients (Werner et al., 2021). Importantly, causality cannot yet be inferred, but the association of poor sleep with both neurodegenerative and inflammatory biomarkers in the same mTBI population should be explored further. This line of exploration could yield novel therapeutic targets, potentially implicating sleep-focused interventions as an accessible therapeutic entry point. Similarly, the development of relevant biomarkers may be useful for patient stratification within clinical trials aimed at novel therapies for mTBI-associated sleep disorders.

### EV-Derived Protein Biomarkers May Confer Unique Utility

EV-based cell-to-cell communication has been linked to pathological mechanisms in a large number of conditions, including TBI (Mustapic et al., 2017; Gurunathan et al., 2019; Paolicelli et al., 2019; Mondello et al., 2020). Whether cytokines are released into peripheral circulation in soluble form or associated with EVs may depend on regulated biological processes and vary with the functional state of biological systems (Fitzgerald et al., 2018). However, the processes that regulate cytokine release after TBI are not well understood. Because the physiologic roles of soluble cytokines derived from the plasma compartment may be different from those of EV-encapsulated/bound cytokines, both were analyzed in the present study. In support of this approach, recent work demonstrates that EV-derived samples may hold additional value compared to plasma cytokine sources. Similar to the present findings, another study of military personnel with a history of chronic mTBI found associations between levels of EV IL-10 and PTSD symptom severity (Devoto et al., 2016). These EV-specific associations may be partially the result of the EV lipid bilayer membrane that shields protein contents from degradation (Winston et al., 2019), thereby increasing signal-to-noise ratios by decreasing the amount of non-specific and non-functional proteins detected in biomarker assays (Chung et al., 2020). In addition to favorable signal strength, increasing evidence suggests that the proteins derived from EVs may serve key functional roles in cell signaling pathways and cell-to-cell transport (Mustapic et al., 2017), perhaps more so than plasma-derived proteins. Our findings support this theory that, although lower in concentration (Supplementary Table S3), EV-derived cytokines may be more clinically relevant for sleep-immune signaling following mTBI. In our population, only EV-derived cytokines were associated with sleep quality (Figures 1, 2; Tables 2, 3). The exception was an unadjusted correlation between PSQI and plasma IL-6 in the mTBI cohort (Table 2), which disappeared once models were adjusted for age, sex, and BMI (Table 3). Future biomarker studies would likely benefit from the inclusion of both plasma and EV-derived samples for the measurement of functionally relevant protein levels.

### Strengths and Limitations

This study presents a preliminary analysis of the association between sleep quality and inflammatory cytokines in warfighters with and without a history of mTBI. Although significant associations were demonstrated within the mTBI cohort, the included population lacked the statistical power for comparison between mTBI and control cohorts. Despite this limitation, we considered the inclusion of a control cohort to be an important aspect for initial exploratory analyses and one that did not diminish the goal of exploring mTBI-relevant associations. Future large-scale studies are needed to determine whether the demonstrated PSQI-cytokine relationships are exclusive to warfighters with mTBI. The current population also consisted exclusively of warfighters; it is unclear whether these results generalize to other TBI populations.

Within this population, we are unable to report on premorbid sleep difficulties or the continuous trajectory of sleep after injury for mTBI participants. The PSQI reports only on sleep complaints experienced during the previous 30 days. Accordingly, reported PSQI-cytokine associations may not be a true representation of long-term sleep difficulties and inflammatory processes occurring continuously in the time since mTBI. However, there is evidence to indicate that sleep symptoms following mTBI may worsen over time (Ma et al., 2019; Lequerica et al., 2020), suggesting that the demonstrated associations are likely gradually building processes.

A significant strength of the current study is the use of both plasma and EV cytokines, providing a novel and important contrast of the relevance of these two protein sources as clinical biomarkers. Additionally, the utilization of a consistent and standardized measure of mTBI through the CENC protocol conferred key diagnostic confidence and relative homogeneity of our mTBI cohort. Furthermore, through this standardized protocol we were able to analyze a cohort of mTBI patients with post-injury timescales averaging nearly a decade. This timescale is significantly longer than relevant prior studies, affording a uniquely valuable preliminary window into prolonged sequelae of mTBI and the associated sleep-immune crosstalk.

## Conclusion

Sleep disorders and poor sleep quality are some of the most prevalent long-term sequelae of mTBI, yet whether sleep quality associates with prolonged inflammatory processes in this population had not previously been explored. Here we demonstrate that poor sleep quality is associated with levels of TNFα and IL-10 cytokines at the chronic stages of mTBI. Clinically, we show that mTBI patients with a diagnosable level of sleep complaints are more likely to exhibit increased IL-10. These findings raise a number of important questions: Is the treatment of sleep complaints an accessible therapeutic entry point for chronic sleep-immune dysregulation following mTBI? Furthermore, can treatments targeted against inflammation improve sleep in mTBI patients? Future prospective studies are needed to characterize the directionality of various aspects of sleep-immune crosstalk following mTBI, as well as the regulatory signaling molecules that may temporally modulate this relationship.

## Data Availability

The raw data supporting the conclusion of this article will be made available by the authors, without undue reservation.
