# Supplementary material for: Poor Sleep Quality is Linked to Elevated Extracellular Vesicle-Associated Inflammatory Cytokines in Warfighters With Chronic Mild Traumatic Brain Injuries
Source: Front Pharmacol. 2022 Jan 27;12:762077. doi: 10.3389/fphar.2021.762077 (PMC8829004; doi:10.3389/fphar.2021.762077)
Supplement: Supplementary file 1 [file DataSheet1.docx]

Supplementary Material

# Supplementary Tables and Figures

## Supplementary Tables

**Supplementary Table 1.** TSG101 protein levels in extracellular vesicle (EV) samples. Levels of the cytosolic protein TSG101, an EV marker, were analyzed in EV samples to confirm presence. Median levels of the protein in control and TBI groups are shown. Missing represents samples with data unavailable.

| **Characteristic** | **Overall**,  N = 182*^1^* | **Control**,  N = 44*^1^* | **TBI**,  N = 138*^1^* | **p-value***^2^* |
| --- | --- | --- | --- | --- |
| TSG101 (pg/mL) | 1,718 (1,235, 2,253) | 1,679 (1,050, 2,150) | 1,746 (1,276, 2,255) | 0.3 |
| Missing | 32 | 7 | 25 |  |
| *^1^*Statistics presented: Median (IQR)  *^2^*Statistical tests performed: Wilcoxon rank-sum test | | | | |

**Supplementary Table 2.** Distribution of protein samples that fell below the lower limit of quantification, organized by cohort and source derivation. Protein values are displayed in pg/mL.

|  | | **# of Samples Below Lower Limit of Quantification (n)** | | **% of Total Samples** | |
| --- | --- | --- | --- | --- | --- |
| **Biomarker** | | Control | mTBI | Control | mTBI |
| EV |  |  |  |  | |
| IL-6 | | 10 | 32 | 26% | 27% |
| IL-10 | | 10 | 35 | 23% | 27% |
| TNFα | | 5 | 35 | 12% | 27% |
| Plasma |  |  |  |  | |
| IL-6 | | 0 | 4 | 0% | 4% |
| IL-10 | | 0 | 0 | 0% | 0% |
| TNFα | | 0 | 0 | 0% | 0% |

**Supplementary Table 3.** Biomarker descriptive statistics according to cohort and presence of clinically significant sleep complaints (PSQI >= 10). Concentrations are reported in pg/mL. * denotes exclusion of samples below lower limit of quantification.

|  | **EV** | | | | | **Plasma** | | | |
| --- | --- | --- | --- | --- | --- | --- | --- | --- | --- |
|  | **mTBI** | | | **Controls** | | **mTBI** | | **Controls** | |
| **Biomarker** | **PSQI < 10** | **PSQI ≥10** | | **PSQI < 10** | **PSQI ≥ 10** | **PSQI < 10** | **PSQI ≥10** | **PSQI < 10** | **PSQI ≥ 10** |
| IL-6 |  | |  |  |  |  |  |  |  |
| Total Samples | 50 | | 67 | 24 | 15 | 46 | 58 | 19 | 12 |
| Samples >  Lower Limit* | 34 | | 51 | 18 | 11 | 43 | 57 | 19 | 12 |
| Median (IQR) | 0.23 (0.60) | | 0.34 (1.48) | 0.15 (0.26) | 0.20 (0.32) | 1.86 (2.03) | 2.60 (2.72) | 1.60 (1.00) | 1.08 (1.62) |
| Median (IQR)* | 0.38 (0.73) | | 0.65 (1.75) | 0.30 (0.21) | 0.22 (0.31) | 1.97 (1.96) | 2.62 (2.73) | 1.60 (1.00) | 1.08 (1.62) |
| IL-10 |  | |  |  |  |  |  |  |  |
| Total Samples | 53 | | 78 | 26 | 18 | 51 | 73 | 25 | 15 |
| Samples > Lower Limit* | 34 | | 62 | 21 | 13 | 51 | 73 | 25 | 15 |
| Median (IQR) | 0.08 (0.16) | | 0.17 (0.19) | 0.18 (0.13) | 0.13 (0.25) | 0.73 (0.53) | 0.98 (0.70) | 0.88 (0.36) | 0.67 (0.58) |
| Median (IQR)* | 0.17 (0.12) | | 0.22 (0.16) | 0.25 (0.13) | 0.23 (0.21) | 0.73 (0.53) | 0.98 (0.70) | 0.88 (0.36) | 0.67 (0.58) |
| TNF⍺ |  | |  |  |  |  |  |  |  |
| Total Samples | 55 | | 77 | 25 | 16 | 42 | 61 | 22 | 11 |
| Samples > Lower Limit* | 38 | | 59 | 22 | 14 | 42 | 61 | 22 | 11 |
| Median (IQR) | 0.41 (0.48) | | 0.54 (0.53) | 0.52 (0.50) | 0.61 (0.34) | 2.94 (1.14) | 2.74 (1.45) | 2.67 (1.45) | 2.85 (0.99) |
| Median (IQR)* | 0.61 (0.40) | | 0.66 (0.39) | 0.56 (0.42) | 0.645 (0.31) | 2.94 (1.14) | 2.74 (1.45) | 2.67 (1.45) | 2.85 (0.99) |

**Supplementary Table 4.** Regression models evaluating number of TBI events as a moderator of PSQI-cytokine (standardized) relationships in mTBI patients. All models control for the effects of age, sex, and BMI. SE represents standard error of the ß coefficient. + denotes *p* < .10; * denotes *p* < .05.

|  | **EV** | | | | **Plasma** | | | |
| --- | --- | --- | --- | --- | --- | --- | --- | --- |
|  | **ß** | **SE** | ***p*-value** | | **ß** | **SE** | ***p*-value** | |
| **IL-6** |  |  |  |  |  |  |  |  |
| PSQI | 0.08 | 0.06 | 0.20 |  | 0.05 | 0.06 | 0.35 |  |
| # TBI | 0.10 | 0.06 | 0.08 | + | 0.09 | 0.06 | 0.11 |  |
| PSQI*# TBI | 0.08 | 0.06 | 0.18 |  | -0.04 | 0.06 | 0.44 |  |
| **IL-10** |  |  |  |  |  |  |  |  |
| PSQI | 0.09 | 0.04 | 0.03 | * | 0.02 | 0.04 | 0.70 |  |
| # TBI | 0.07 | 0.04 | 0.12 |  | -0.03 | 0.04 | 0.51 |  |
| PSQI*# TBI | 0.05 | 0.04 | 0.27 |  | 0.02 | 0.04 | 0.52 |  |
| **TNF⍺** |  |  |  |  |  |  |  |  |
| PSQI | 0.06 | 0.03 | 0.02 | * | -0.01 | 0.03 | 0.74 |  |
| # TBI | 0.03 | 0.03 | 0.21 |  | -0.01 | 0.03 | 0.59 |  |
| PSQI*# TBI | 0.01 | 0.03 | 0.59 |  | 0.01 | 0.03 | 0.59 |  |

**Supplementary Table 5.** Regression models evaluating cohort as a moderator of PSQI-biomarker (standardized) relationships. All models control for the effects of age, sex, and BMI. SE represents standard error of the ß coefficient. + denotes *p* < .10; * denotes *p* < .05.

|  | **EV** | | | | **Plasma** | | |  |
| --- | --- | --- | --- | --- | --- | --- | --- | --- |
|  | **ß** | **SE** | ***p*-value** | | **ß** | **SE** | ***p*-value** | |
| **IL-6** |  |  |  |  |  |  |  |  |
| PSQI | 0.09 | 0.05 | 0.07 | + | 0.04 | 0.05 | 0.36 |  |
| Cohort | -0.07 | 0.05 | 0.17 |  | -0.05 | 0.05 | 0.33 |  |
| PSQI*Cohort | 0.09 | 0.05 | 0.05 | + | -0.03 | 0.05 | 0.58 |  |
| **IL-10** |  |  |  |  |  |  |  |  |
| PSQI | 0.08 | 0.04 | 0.04 | * | 0.00 | 0.03 | 0.93 |  |
| Cohort | 0.04 | 0.04 | 0.25 |  | -0.02 | 0.03 | 0.63 |  |
| PSQI*Cohort | 0.02 | 0.04 | 0.67 |  | 0.01 | 0.03 | 0.64 |  |
| **TNF⍺** |  |  |  |  |  |  |  |  |
| PSQI | 0.06 | 0.02 | 0.01 | * | -0.01 | 0.02 | 0.54 |  |
| Cohort | 0.01 | 0.02 | 0.65 |  | 0.00 | 0.02 | 0.91 |  |
| PSQI*Cohort | 0.03 | 0.02 | 0.25 |  | 0.01 | 0.02 | 0.60 |  |

**Supplementary Table 6.** Regression models evaluating number of TBIs as a moderator of sleeper type-biomarker (standardized) relationships. All models control for the effects of age, sex, and BMI. SE represents standard error of the ß coefficient. + denotes *p* < .10; * denotes *p* < .05; ** denotes *p* < .01.

|  | **EV** | | | | **Plasma** | | | |
| --- | --- | --- | --- | --- | --- | --- | --- | --- |
|  | **ß** | **SE** | ***p*-value** | | **ß** | **SE** | ***p*-value** | |
| **IL-6** |  |  |  |  |  |  |  |  |
| Sleeper Type | -0.06 | 0.06 | 0.27 |  | -0.08 | 0.05 | 0.12 |  |
| # TBI | 0.11 | 0.06 | 0.07 | + | 0.09 | 0.05 | 0.10 |  |
| Sleeper Type*# TBI | 0.09 | 0.06 | 0.15 |  | -0.04 | 0.06 | 0.49 |  |
| **IL-10** |  |  |  |  |  |  |  |  |
| Sleeper Type | -0.11 | 0.04 | 0.01 | ** | -0.02 | 0.04 | 0.54 |  |
| # TBI | 0.08 | 0.04 | 0.05 | + | -0.02 | 0.04 | 0.63 |  |
| Sleeper Type*# TBI | 0.05 | 0.04 | 0.26 |  | 0.03 | 0.04 | 0.45 |  |
| **TNF⍺** |  |  |  |  |  |  |  |  |
| Sleeper Type | -0.04 | 0.03 | 0.10 |  | -0.01 | 0.02 | 0.69 |  |
| # TBI | 0.05 | 0.03 | 0.08 | + | -0.01 | 0.02 | 0.73 |  |
| Sleeper Type*# TBI | 0.02 | 0.03 | 0.54 |  | 0.02 | 0.02 | 0.48 |  |

## Supplementary Figures

##
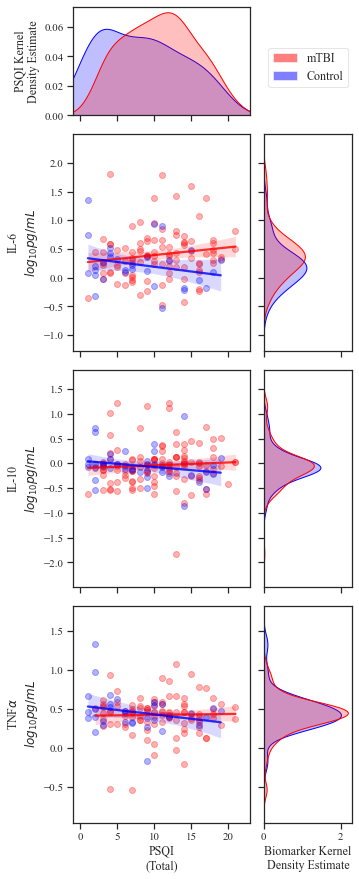


**Supplementary Figure 1.** **Correlations between PSQI score and plasma-derived inflammatory biomarker concentrations.** **Main plots)** Scatterplots of PSQI versus plasma biomarker concentration. Lines represent linear best fit with 95% confidence intervals. Plasma IL-6 levels significantly correlated with PSQI score in mTBI patients. **Marginal plots)** Kernel density estimates representing the distribution of PSQI scores (top) and biomarker concentrations (right) for each cohort. Blue = control patients; Red = mTBI patients. For clarity, data are visualized without values below lower limits of quantification.
